# Conceptual Advances in Control of Inflammation by the RNA-Binding Protein Tristetraprolin

**DOI:** 10.3389/fimmu.2021.751313

**Published:** 2021-09-17

**Authors:** Pavel Kovarik, Annika Bestehorn, Jeanne Fesselet

**Affiliations:** Max Perutz Labs, University of Vienna, Vienna Biocenter (VBC), Vienna, Austria

**Keywords:** tristetraprolin (TTP), zinc finger protein 36 (Zfp36), RNA binding protein, mRNA stability/decay, inflammation, immune system, immune homeostasis

## Abstract

Regulated changes in mRNA stability are critical drivers of gene expression adaptations to immunological cues. mRNA stability is controlled mainly by RNA-binding proteins (RBPs) which can directly cleave mRNA but more often act as adaptors for the recruitment of the RNA-degradation machinery. One of the most prominent RBPs with regulatory roles in the immune system is tristetraprolin (TTP). TTP targets mainly inflammation-associated mRNAs for degradation and is indispensable for the resolution of inflammation as well as the maintenance of immune homeostasis. Recent advances in the transcriptome-wide knowledge of mRNA expression and decay rates together with TTP binding sites in the target mRNAs revealed important limitations in our understanding of molecular mechanisms of TTP action. Such orthogonal analyses lead to the discovery that TTP binding destabilizes some bound mRNAs but not others in the same cell. Moreover, comparisons of various immune cells indicated that an mRNA can be destabilized by TTP in one cell type while it remains stable in a different cell linage despite the presence of TTP. The action of TTP extends from mRNA destabilization to inhibition of translation in a subset of targets. This article will discuss these unexpected context-dependent functions and their implications for the regulation of immune responses. Attention will be also payed to new insights into the role of TTP in physiology and tissue homeostasis.

## Introduction

It is now well accepted that regulation of mRNA stability by RNA-binding proteins (RBPs) is indispensable for healthy immune responses. RBPs orchestrate the immune system by modulating gene expression through mRNA destabilization or stabilization, or by controlling translation (1–3). Although this basic knowledge is established, many important questions remain unresolved. These include mechanistic explanations of the phenotype caused by an RBP deletion in mice and the selective functions of RBPs in specific cell types despite ubiquitous expression. Improved models of the molecular mechanisms of RBP action are needed to answer the open questions. These models will likely abandon the linear schemes in which RBP binding to a target mRNA inevitably results in a canonical consequence, e.g. mRNA decay. The aim of this review is to provide a framework for updated models of RBP action in immune responses.

The history of mRNA decay research, both at the level of mechanisms and functions, is tightly connected to the immune system. The first evidence that selective mRNA degradation is driven by a cis-acting element was reported for the mRNA encoding the granulocyte/monocyte growth factor GM-CSF (4). This study established that an adenylate-uridylate-rich element (AU-rich element; ARE) in the 3’ untranslated region (3’ UTR) of the GM-CSF mRNA (encoded by the *CSF2* gene) confers mRNA instability if introduced into the 3’ UTR of a stable mRNA. The autonomous effect of AREs on mRNA stability has been subsequently documented for many other mRNAs. The key role of ARE-dependent mRNA decay *in vivo* was revealed by the deletion of the ARE in the mouse *Tnf* gene which resulted in a spontaneous development of gut and joint inflammation (5). However, genome sequencing and transcriptome-wide mRNA stability assays indicated that the initial model of an autonomous function of 3’ UTR-located AREs in mRNA destabilization was too simple. Approximately 20% of human genes contain AREs in their 3’ UTRs, yet most of the corresponding mRNAs are stable (6, 7). The medium half-life of mRNA in human HepG3 cells is approximately 10 h with mRNAs of metabolic genes having on average the highest half-lives (6). In comparison, inflammation-associated mRNAs belong to those with the shortest average half-lives. For illustration, the decay rate of *TNF* mRNA is in the range of 20 – 40 min, depending on the cell type and stimulus (8, 9). Although inflammation-induced mRNAs are enriched in AREs, it is now accepted that the presence of an ARE is not sufficient to destabilize the mRNA. Hence, new and more comprehensive models of regulation of mRNA decay by cis-acting elements are needed.

## Mechanisms of RBP-Driven Changes in mRNA Stability

mRNA-destabilizing RBPs bind and facilitate the target mRNA degradation in two ways, depending on the properties of the particular RBP (1, 2, 10, 11). One class of RBPs possesses an endonuclease activity which allows the RBP to cleave the target mRNA and generate ends devoid of the 5’ m^7^G cap and the 3’ poly(A) tail. These unprotected ends serve as substrates for exonucleases which process the mRNA in 3’ – 5’ direction *via* the exosome and 5’ - 3’ direction *via* XRN1 (12, 13). The best characterized endonucleolytic RBP relevant for the immune system is Regnase-1 (gene name Zc3h12a) which destabilizes mRNAs of transcription factors and cytokines such as Icos, Ox40, c-Rel, IL-2 and IL-6 (14). Regnase-1-deficient mice show severe systemic inflammation associated with T and B cell activation. The phenotype is largely recapitulated by a T cell-specific deletion (14). The second class of RBPs destabilize the target mRNA by promoting the recruitment of the CCR4-NOT deadenylase and the DCP1/DCP2-containing decapping complexes (15, 16). A number of RBPs in this class are known to regulate the immune system. Tristetraprolin (TTP), as one of the most prominent members, will be described in detail below. Other well characterized members are Roquin-1 (gene name *Rc3h1*), Roquin-2 (*Rc3h2*), Zfp36l1, Zfp36l2 and Auf1. The Roquin proteins redundantly target the mRNAs of Icos and Ox40 to control T cell activation. Deletion of both Roquin-1 and Roquin-2 genes specifically in CD4 T cells results in an autoimmune phenotype resembling systemic lupus erythematosus while deletion of the single genes remains without severe consequences (17). The proteins Zfp36l1 and Zfp36l2 are members of the TTP family but, in contrast to TTP, have more pleotropic functions as demonstrated by embryonic or postnatal lethality of the respective knockouts in mice (18, 19). Zfp36l1 and Zfp36l2 are involved in the regulation of immune system in multiple ways. They control the expression of proliferative cell cycle regulators during B and T cell development: double deletion of Zfp36l1 and Zfp36l2 in T cells results in lymphopenia and malignant transformation of immature CD8 T cells while similar deletion in pro-B cells causes a block in B cell development owing to a failure in entering quiescence hence genome safeguarding prior to VDJ recombination (20, 21). The protein Auf1 exhibits anti-inflammatory functions by promoting the degradation of cytokine mRNAs as revealed by the hypersensitivity of Auf1-deficient mice to endotoxic shock (22). Auf1 has been subsequently found to regulate many other processes in addition to immune responses including telomere maintenance and muscle regeneration (23, 24).

mRNA-stabilizing RBPs are less well understood and their functions are more pleiotropic as compared to the destabilizing RBPs such as TTP. The general opinion is that mRNA-stabilizing RBPs act by preventing the destabilizing proteins from binding to the target. As a consequence, the target mRNAs are more stable and/or more efficiently translated. mRNA-stabilizing RBPs regulating immune responses include HuR (gene name *Elavl1*) and Arid5a. Deletion of HuR or Arid5a in mice resulted in increased resistance to experimental autoimmune encephalomyelitis (25, 26). Furthermore, HuR is required for antibody production by B cells (27). HuR is involved in regulation of other processes including liver metabolism, cell proliferation and cancer (28–30).

## RNA Binding of RBPs Controlling mRNA Stability

RBPs bind to RNA through interactions of their RNA-binding domains with specific sequences or defined structural elements in the target mRNA. The most frequent RNA-binding domain in the immunoregulatory RBPs is the C3H1 (Cys-Cys-Cys-His) zinc finger domain present for example in TTP, Zfp36l1 and Zfp36l2 (2, 31). Regnase and Roquin contain a C3H1 zinc finger and an additional RNA-binding domain: a PIN domain and a ROQ domain, respectively (32, 33). Auf1 and HuR bind to RNA through the RNA recognition motif (RRM) domains which occur in 2 or 3 repeats in these proteins (34, 35). Arid5a interacts with RNA *via* an ARID domain which is known to recognize DNA in other ARID domain-containing proteins (26, 36).

The target site in the RNA is defined by the RNA-binding domain. The C3H1 zinc finger present in TTP, Zfp36l1 and Zfp36l2 binds preferentially to AREs with the core sequence UAUUUAU although divergent target sites have been identified as well (9, 21, 37). A preference for AU-rich sequences shows also the ARID domain of Arid5a (26). The RRM domain of Auf1 recognizes U- and GU-rich stretches and, albeit less frequently, AREs (38). The RRM motif of HuR prefers U-rich sequences (9, 39, 40). The preference of these binding domains for AREs or U-rich sequences reflects the unstructured nature of such sequences: AREs and U-rich sequences in general do not adopt a secondary structure. In contrast, Regnase and Roquin bind to RNAs exhibiting stem-loop folds with the loop part formed by three bases with a pyrimidine–purine–pyrimidine sequence while the stem is more variable both in length (5 – 8 bases in each half of the stem) and sequence (41, 42).

Transcriptome-wide binding assays revealed that most of these RBPs bind frequently to 3’ UTR and, unexpectedly, introns (9, 21, 38–40). Binding to introns regulates splicing in case of HuR (40, 43). However, it appears that functional interactions are largely confined to elements located in the 3’ UTRs as intronic binding in general does not result in changes in stability or splicing of the transcript.

## The TTP Protein Family: Evolutionary Conserved RBPs With Diverse Functions From Yeast to Mammals

TTP contains an RNA-binding domain formed by a characteristic tandem C3H1 zinc finger in the middle part and protein-protein interaction domains at the N- and C-termini (31). The tandem zinc finger and the overall domain structure are conserved in similar RBPs from yeast to plants and mammals hence these RBPs constitute the TTP protein family (44). Interestingly, no TTP protein members are found in birds despite their presence in reptiles (44). Although all these proteins facilitate mRNA degradation their functions in cells and/or organisms are diverse. For example, the yeast TTP family member Cth2 regulates mRNA stability upon iron deficiency while the Xenopus TTP proteins act during embryonic development and the *C. elegans* homologues are required for meiosis and oocyte production (45–47). Humans contain three TTP family members (Zfp36, Zfp36l1 and Zfp36l2) and mice express the Zfp36l3 member in addition. Much of what we now know about the functions of the TTP protein family has been learned from knockouts in mice carried out by the Blackshear laboratory. Deletion of Zfp36l1 (also known as BRF1 and TIS11b) is embryonic lethal because of failure in umbilical circulation resulting from absent fusion of the allantois with the chorion (18). Mice lacking Zfp36l2 (also known as BRF2 and TIS11D) die within a few weeks after birth due to a marked deficiency in hematopoiesis (19). Zfp36l3 is a paternally imprinted X chromosome gene which is likely involved in regulation of iron metabolism in the placenta; Zfp36l3 deletion results in decreased neonatal survival rates without obvious morphological aberrances in surviving offspring (48).

## TTP: a TTP Family Member With Unique Selectivity for the Regulation of Immune Responses

TTP (Zfp36) is an outstanding member of the TTP family as its function is remarkably specific and related to the regulation of immune responses. TTP knockout in mice results in systemic inflammation characterized by arthritis, dermatitis, conjunctivitis and cachexia (49). This so called TTP deficiency syndrome develops within approximately 8 weeks of birth and progressively worsens leading to death of most animals at around 6-8 months of age. TTP-deficient mice do not show any developmental abnormalities or health defects at birth; the mice are not fertile presumably owing to their poor health (49). The TTP deficiency syndrome was shown to be dependent on TNF signaling and mechanistically explained by increased stability of *Tnf* mRNA (49, 50). Subsequent studies established that the inflammatory disease of TTP-deficient mice is caused, albeit to variable extent, by increased stability of other cytokine and chemokine mRNAs as well, notably *Il23*, *Ccl3*, *Il1a* and *Il1b* mRNAs (51–53).

Given this multiple evidence for its indispensable role in the immune system, it comes with no surprise that TTP has become one of the best studied RBPs. However, many important questions remain open. For example, it is not well understood which cell types drive the inflammatory disease in TTP-deficient mice. Mice bearing LysM-Cre-mediated TTP deletion in the myeloid compartment are healthy which is unexpected given that myeloid cells are cells with arguably the highest TTP expression (54, 55). Although these mice exhibit lethal hypersensitivity to endotoxic shock, the absence of a spontaneous inflammation suggests that deletion of TTP in myeloid cells alone is not sufficient to cause the TTP deficiency syndrome. Similarly, mice with CD11c-Cre-driven deletion of TTP in dendritic cells remain without a spontaneous phenotype (56). Surprisingly, systemic inflammation arises upon deletion of TTP in keratinocytes (56). The inflammatory disease in these mice develops from psoriasis-like focal skin lesions containing neutrophilic infiltrates, indicating that persistent local inflammation can become systemic with time. The model of keratinocyte-specific TTP deletion suggests that TTP expression is particularly critical in barrier tissues, i.e. tissues constantly exposed to environmental cues. However, it is remarkable that the full-body TTP knockout mice remain without pathology in the intestinal or lung epithelium, i.e. the most prominent mucosal barriers. The absence of mucosal inflammation in TTP-deficient mice suggests that TTP has more complex roles in these tissues. Such functional complexity is supported by findings showing that the lack of intestinal pathology in TTP knockout mice is associated with a local expansion of regulatory T cells (57). Moreover, Villin-Cre-driven TTP deletion in intestinal epithelial cells increases the resistance against dextran sulfate-induced colitis suggesting, that the lack of TTP might enhance the robustness of the intestinal barrier (58). Although the mechanism is yet to be determined, the improved mucosal barrier might be caused by accelerated tissue regeneration since these mice exhibit higher numbers of Goblet cells. These findings suggest that the absence of TTP augments proliferation signals that are commonly associated with inflammatory conditions. In agreement, skin inflammation caused by TTP deficiency in keratinocytes promotes tumorigenesis that appears to be causally associated with overproduction of the growth factor amphiregulin (59). Consistently, amphiregulin mRNA is a TTP target. However, TTP deficiency can cause increased cell numbers also by means of decreased apoptosis as shown for TTP-deficient neutrophils: neutrophils devoid of TTP express higher levels of the TTP target *Mcl1* mRNA which codes for an anti-apoptotic factor particularly relevant for neutrophils (60). Interestingly, this effect pertains only to immunostimulated (e.g. pathogen-engaged) neutrophils, not to the circulating dormant neutrophil pool.

Cumulatively, the available animal models of TTP deficiency clearly indicate that the major function of TTP is to control the immune response. Although TTP restricts cell numbers in some cases, this function is also largely related to control of inflammation: (i) by ameliorating inflammation TTP prevents the expression of inflammation-associated growth factors or anti-apoptotic proteins, (ii) TTP directly targets the mRNAs of several inflammation-associated growth or anti-apoptotic factors. More studies directly investigating cells from tissues are needed to complete our understanding of TTP effects *in vivo*.

## mRNA Destabilization by TTP

TTP promotes mRNA decay through the recruitment of the CCR4-NOT deadenylase and the DCP1/DCP2 decapping complexes to the bound target. The N- and C-termini of TTP represent the protein-protein interaction domains in this process. The CCR4-NOT deadenylase complex interacts with the N- and C-terminal domains with the CNOT1 subunit being directly involved in binding to TTP (61–64). The DCP1 and DCP2 decapping protein complexes interact with the N-terminal TTP domain (62). Following decapping and deadenylation, the target mRNA is degraded through the 5′-3′ exonuclease Xrn1 and the 3′-5′ exonuclease of the exosome, respectively. The identification of these interactions suggested that TTP-mediated mRNA degradation is governed by a protein recruitment cascade. However, this model does not explain why many TTP-bound RNAs (including mRNAs and introns) are stable as shown by more recent studies (9, 65–67). The surprising findings of these studies delineate that the process of mRNA destabilization by TTP is more complex and dependent on yet unidentified regulatory mechanisms.

## TTP Binding to RNA

**Figure 1:**
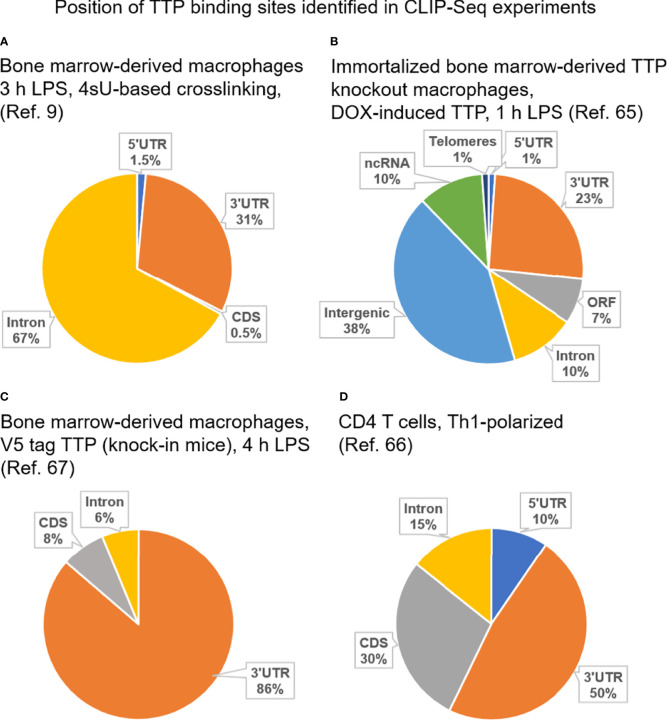
Position of TTP binding sites identified in reported CLIP-Seq experiments employing immune cells. **(A)** CLIP-Seq experiment carried out using bone marrow-derived macrophages isolated from wild type mice (i.e. expressing solely endogenous TTP). Cells were stimulated for 3 h with LPS prior to CLIP-Seq which was based on thiouridine (4sU)-mediated crosslinking allowing crosslinking with 365 nm UV, i.e. mild conditions [(9)]. **(B)** CLIP-Seq experiment performed using immortalized bone marrow-derived macrophages isolated from TTP knockout mice and engineered to express doxycycline-inducible TTP. Cells were treated with doxycycline and stimulated for 1 h with LPS prior to CLIP-Seq [(65)]. **(C)** CLIP-Seq experiment carried out using bone marrow-derived macrophages isolated from mice expressing V5-tagged TTP from the endogenous locus (knock-in mice). Cells were stimulated for 4 h with LPS prior to CLIP-Seq [(67)]. **(D)** CLIP-Seq experiment carried out using CD4+ T cells from wild type mice [(i.e. expressing solely endogenous TTP)]. CD4+ T cells were polarized under Th1 conditions prior to CLIP-Seq [(66)]. 3’ UTR, 3’ untranslated region; 5’ UTR, 5’ untranslated region; CDS, coding sequence; ORF, open reading frame; ncRNA, non-coding RNA; DOX, doxycycline.

Both zinc finger domains are required for interaction of TTP with RNA as mutation of either of them abrogates RNA binding (68). Moreover, mutation of the first zinc finger in the TTP locus in mice phenocopied the complete TTP deletion (69). This was a significant finding as it definitively proved that the function of TTP is entirely dependent on its RNA binding activity. Initial characterization of the motif recognized by TTP focused on the TNF mRNA, the first known TTP target: the motif is a 9-mer with the sequence UUAUUUAUU which is repeated several times in the TNF 3’ UTR (37, 70). Subsequent analysis of RNAs enriched in RNA immunoprecipitation assays suggested that TTP binds to AREs also in other target mRNAs (71). A precise genome-wide mapping of target sequences was generated by several CLIP-Seq (cross-linking immunoprecipitation-high-throughput sequencing) studies employing immune cells. Although these nucleotide resolution analyses confirmed the preference of TTP for the UAUUUAU sequence, they also provided several unexpected findings (9, 65–67). The studies showed that TTP binds also to sites that were divergent from the canonical TTP binding sequence as visualized in the searchable TTP Atlas (https://ttp-atlas.univie.ac.at) (9). Moreover, TTP binding was not limited to 3’ UTRs but was detected at sites located in 5’ UTRs, coding sequences and introns as well (**Figure 1**). Particularly striking was the high incidence of TTP binding to introns. Although the number of identified intronic binding sites was dependent on the CLIP-Seq method, the peak finding algorithm and experimental cell system, the studies convincingly established that TTP interacts with pre-mRNA in addition to mRNA. This finding implies that TTP can engage RNA interactions in the nucleus. The biological significance of the intronic binding remains to be determined as no effects on splicing or stability of the intron-bound RNA has so far been observed (9). Given the high frequency of TTP binding to introns it is possible that introns act as sponge to titrate away TTP molecules. This mechanism was reported for circular RNAs that function as sponge molecules for micro RNAs (72, 73). Similar to intronic binding, it is currently unclear whether interactions of TTP with 5’ UTRs or coding sequences entail changes in RNA processing.

The CLIP-Seq data show that functional TTP bindings sites are located in 3' UTR. Remarkably, binding of TTP to 3’ UTR does not always cause destabilization of the target mRNA, as revealed in recent studies. This enigmatic and probably significant property of TTP is discussed further below.

## Regulation of TTP

TTP function is regulated in multiple ways with many of them remaining poorly understood. Moreover, it is likely that some key regulatory events are still not known. Comprehensive knowledge about the regulation of TTP is critical for our understanding of the remarkably selective function of TTP in the immune system and for the control of inflammation in general. TTP is regulated at the level of transcription, mRNA stability, protein stability and by posttranslational modifications (31, 74–76). As far as we can say, all these mechanisms are critical for the appropriate extent, timing and selectivity of TTP-driven mRNA degradation. They act in concert to allow the immune system launching an efficient but not exaggerated inflammatory response.

TTP mRNA levels are low under steady state conditions but dramatically induced in response to inflammatory stimuli which are mostly associated with stress signaling. The increase in TTP mRNA levels is achieved mostly by transcriptional induction and to some extent also through mRNA stabilization. As an immediate early gene, TTP is transcriptionally activated rapidly after stimulation. The activation signals include growth factors, cytokines such as TNF, IL-4, IL-10, or IFN-γ, and bacterial products e.g. LPS (50, 77–80). The transcription factors involved in the transcriptional upregulation were characterized in few instances: IFNs, IL-10 and IL-4 drive TTP expression through STAT1, STAT3 and STAT6, respectively (78–80). STAT1 employs a GAS (Gamma interferon activation site) element which is conserved in the TTP promoter in mice and humans (78). This GAS element is likely involved also in response to IL-4 and IL-10. The activating signals often synergize to achieve maximal induction of TTP (78, 79).

Stabilization of TTP mRNA by p38 MAPK signaling contributes to induction of TTP expression (81). TTP mRNA contains AREs which interact with TTP protein suggesting that autoregulation is the mechanism underlying the low TTP mRNA stability. TTP mRNA is indeed moderately more stable in TTP knock-in mice expressing the zinc finger-inactivated mutant (69).

A central aspect of the regulation of TTP levels in cells is the control of TTP protein stability. TTP is continuously degraded in a proteasome-dependent way which appears to proceed without ubiquitination and is likely to involve the intrinsically unfolded N- and/or C-terminal domains (82, 83). The mechanism of this important process is not resolved and its elucidation would significantly advance our understanding of protein degradation in general. TTP protein stability increases by orders of magnitude upon phosphorylation of S52 and S178 (in mouse coordinates) (82, 84). Phosphorylation of these two residues is brought about by MK2, a kinase that is activated by p38 MAPK. Although the p38 MAPK/MK2-driven phosphorylation of S52 and S178 increases TTP protein stability and thereby positively regulates TTP levels, it inhibits the mRNA-destabilization activity of TTP. This phosphorylation-dependent TTP inhibition probably results from a combination of several processes: (i) S52 and S178 phosphorylation causes association of TTP with 14-3-3 proteins thereby preventing relocation of TTP to stress granules and processing bodies, (ii) 14-3-3 protein binding promotes export of TTP from the nucleus, (iii) S52 and S178 phosphorylation decreases association of TTP with the CCR4-NOT deadenylase, and (iv) MK2-dependent TTP phosphorylation diminishes TTP binding to RNA (85–88). Although the mechanistic details of the function of S52 and S178 phosphorylation are not fully understood, the biological consequences have been convincingly revealed by generation of double knock-in mice bearing S52A and S178A mutations in the TTP locus (89). These mice are unable to express high TTP protein levels, consistent with a rapid TTP protein degradation. Nevertheless, the mice are protected against LPS-induced systemic inflammation indicating that the S52A/S178A mutant acts as hyperactive TTP *in vivo*. The double knock-in mouse confirmed the previously proposed model of TTP function according to which p38 MAPK leads to accumulation of inactive (i.e. phosphorylated) TTP in the initial phase of inflammation. Later, i.e. in the resolution phase of inflammation, the gradual decrease of p38 MAPK activity releases TTP from its inhibited state thereby facilitating degradation of TTP target mRNAs (9, 55). In parallel, the diminishing phosphorylation accelerates proteasomal degradation of TTP rendering the cells responsive to a new inflammatory stimulus.

TTP contains more than 30 phosphorylation sites out of which only S52 and S178 have been functionally annotated in cells and animals (90). A recent quest for a better understanding of TTP phosphorylation has revealed MK2-dependent phosphorylation of T84, S85, T250, and S316 out of which the phosphorylation of S316 is the most robust one (91). Notably, S316 phosphorylation in not involved in regulation of TTP protein stability; instead, it appears to regulate interactions of TTP with the translation inhibition proteins (91). It will be exciting to see the progress in functional characterization of other phosphorylation sites as they likely impinge on TTP in unexpected ways.

## To Degrade or Not to Degrade the Bound mRNA?

**Figure 2:**
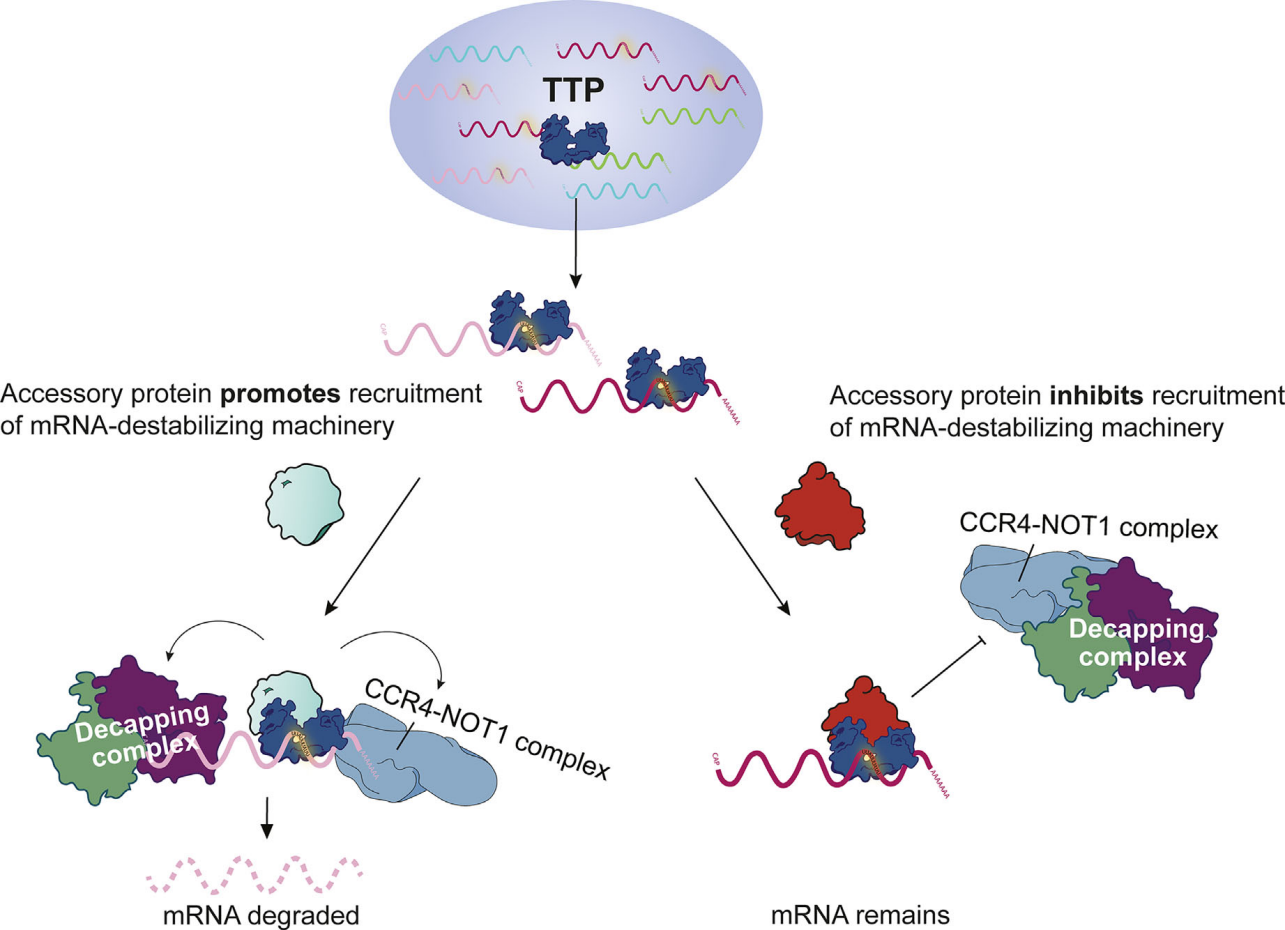
Model of functional and silent binding of TTP to mRNA. Model of possible mechanisms explaining how mRNA-bound TTP destabilizes some mRNAs but not others.

Transcriptome-wide mRNA stability studies coupled to CLIP-Seq analyses revealed that, surprisingly, TTP does not always cause degradation of the bound target. This has been convincingly demonstrated by employing bone marrow-derived macrophages expressing solely endogenous TTP (9). The study showed that 71% of mRNAs bound by TTP in their 3’ UTR are stable. A similar conclusion was drawn from CLIP-Seq and mRNA stability assays in HEK293 cells overexpressing TTP (92). These observations were supported by other CLIP-Seq studies employing primary cells (i.e. cells not overexpressing TTP) although the evidence was indirect as it was based on differential expression analysis (RNA-Seq) but not on transcriptome-wide mRNA stability assessments (66, 67). These unexpected results indicate that a more complex model of TTP action needs to be developed. The model will probably involve proteins acting in cis with TTP which prevent recruitment of the RNA degradation machinery to stable transcripts or facilitate such recruitment to unstable transcripts (**Figure 2**). This new concept could also involve yet uncharacterized TTP phosphorylation events; in this scenario a particular phosphorylation (activating or inactivating) would occur only on certain target mRNAs and/or subcellular locations. The advanced concept of TTP function might also consider a recently reported hypothesis that TTP stabilizes mRNA under certain circumstances: The dramatic induction of TTP following an inflammatory stimulus was proposed to generate a pool of free TTP that sequesters the RNA degradation machinery thereby preventing mRNA decay, but a direct evidence for this hypothesis was not provided (93).

Related to the question of whether TTP destabilizes or not a specific subset of bound mRNAs are ribosome profiling data showing that TTP can affect mRNA stability but also inhibit translation (65–67). Particularly conclusive were studies employing primary macrophages and T cells, i.e. experimental systems expressing solely endogenous and naturally regulated TTP (66, 67). All these studies revealed transcript-selective effects of TTP on translation: some TTP target mRNAs were less abundant at polysomes while others were not depleted from polysomes. Negative regulation of translation has been also implicated in control of inflammatory gene expression in tumor-associated macrophages (94). All these data are consistent with an updated model of TTP function in which cis-acting and transcript-specific RBPs determine the final consequence of TTP binding to the target mRNA (**Figure 2**). This updated model is supported by the finding that TTP binds to the cytoplasmic poly(A)-binding protein and that such interactions are required for inhibition of translation by TTP in primary macrophages (67). The model will become more complex once data on tissue/cell type-specific functions of TTP are included. First data on biologically relevant cell type-specific effects of TTP have only recently become available: The mRNA coding for the IL-1β cytokine (*Il1b* mRNA) is destabilized by TTP in bone marrow-derived dendritic cells but not in bone marrow-derived macrophages despite strong binding to TTP (53). The regulation of *Il1b* expression by TTP is important *in vivo* as TTP-deficient mice show higher *Il1b* mRNA levels in several tissues. Moreover, genetic inactivation of IL-1 signaling in TTP-deficient animals ameliorates the TTP deficiency syndrome (53).

The extent of cell type-specific regulation of TTP activity can be indirectly estimated from a number of RNA-seq studies comparing mRNA levels in wild-type *versus* TTP knockout cells. For example, the levels of *Tnf* mRNA, the bona fide TTP target, are comparable in wild-type and TTP-deficient T cells, suggesting that TTP does not target Tnf mRNA for degradation in T cells in contrast to most other cell types (66). Similarly, *Il6* mRNA, which is known to be bound and destabilized by TTP in bone marrow-derived macrophages (BMDMs) (55), is more highly expressed also in TTP-deficient dendritic cells (upon 3 h or 6 h LPS stimulation) and T cells (upon 4 h activation) but not in peritoneal neutrophils (53, 60). Reported RNA-seq expression data for selected TTP targets in primary immune cells (BMDMs, BMDCs, peritoneal neutrophils and T cells) from wild-type and TTP-deficient mice are summarized in **Table 1**. These data convincingly visualize that a comparison of mRNA levels does not provide a definitive information about the destabilization of a particular mRNA by TTP since indirect (possibly cell type-specific) effects of transcription can mask differences in mRNA stability. A good example is *Il6* mRNA in T cells: while a short (4 h) activation results in 50% higher *Il6* mRNA levels in TTP-deficient T cells as compared to controls, consistent with *Il6* mRNA being destabilized by TTP, a 3-day activation causes TTP-deficient T cells to express 15% less *Il6* mRNA than the control cells [**Table 1** and (66)]. Thus, mRNA stability assays combined, whenever possible, with TTP binding analyses are required when defining a TTP target in a given cell type. A combination of transcriptome-wide mRNA stability and TTP binding assays has been so far reported only for BMDMs so that a comprehensive TTP target collection (searchable at https://ttp-atlas.univie.ac.at/) is available only for this cell type (9). Future studies should include transcriptome-wide mRNA stability analyses in other cell types.

**Table 1:** Expression data (FPKMs or counts) of selected TTP targets in different immune cell types from wild type and TTP-KO mice.

Gene_ID	Gene_name	BMDM, 3 h LPS, FPKM		BMDM, 6 h LPS, FPKM		BMDC, 3 h LPS, FPKM		BMDC, 6 h LPS, FPKM		Peritoneal neutrophils, 4 h LPS, FPKM		T cells, 4 h activation, counts		T cells, 3 day activation, counts	
		Wild-type	TTP-KO	Wild-type	TTP-KO	Wild-type	TTP-KO	Wild-type	TTP-KO	Wild-type	TTP-KO	Wild-type	TTP-KO	Wild-type	TTP-KO
ENSMUSG00000029378	Areg	ND	ND	ND	ND	3.93	8.12	0.17	0.88	12.05	9.68	89.50	51.75	217.00	87.00
ENSMUSG00000000982	Ccl3	6937.76	9117.56	4183.66	16518.37	1260.15	4309.57	814.67	4136.92	8134.45	9617.97	22.50	22.00	56187.67	21074.00
ENSMUSG00000018930	Ccl4	9008.07	10206.05	4422.39	6764.60	652.02	1358.16	234.16	971.93	4055.42	3860.08	201.25	210.00	134778.33	80107.67
ENSMUSG00000029380	Cxcl1	629.10	1589.36	24.27	443.73	309.38	692.45	127.86	374.72	1900.60	2273.85	1.00	3.50	0.00	0.00
ENSMUSG00000034855	Cxcl10	11244.27	10914.54	10272.14	9763.24	494.36	1296.76	435.80	1888.26	57.05	51.66	486.00	475.50	12.00	10.67
ENSMUSG00000058427	Cxcl2	1563.82	5658.09	179.03	3428.48	862.66	3066.80	325.44	2415.03	27007.28	45947.78	8.50	14.25	260.33	60.33
ENSMUSG00000029379	Cxcl3	384.68	351.67	35.31	230.81	948.92	1404.63	536.80	1536.96	3174.43	3472.62	13.50	26.50	808.67	422.67
ENSMUSG00000003541	Ier3	173.04	335.38	215.25	358.83	27.72	69.97	31.33	77.18	999.89	1081.84	106.00	190.25	3273.00	12006.67
ENSMUSG00000016529	Il10	71.76	171.35	129.47	680.55	0.67	5.57	0.22	2.99	347.01	719.35	3.75	6.25	105982.33	149397.33
ENSMUSG00000027399	Il1a	32.43	60.61	13.80	28.18	286.92	737.09	173.03	547.67	3642.63	4825.90	17.75	29.25	95.00	18.33
ENSMUSG00000027398	Il1b	138.34	150.00	70.07	82.29	412.66	1241.13	259.85	940.15	9785.50	14043.60	56.50	96.50	0.00	0.00
ENSMUSG00000025746	Il6	72.51	62.09	35.80	76.62	293.93	629.44	317.59	684.42	324.30	201.17	50.00	76.25	57.00	42.33
ENSMUSG00000032487	Ptgs2 (or Cox2)	206.85	155.98	1396.01	1288.74	1.09	3.96	3.14	12.91	6719.78	6566.11	5.25	9.50	26.00	49.67
ENSMUSG00000020826	Nos2	309.54	357.49	469.47	849.94	79.64	165.24	44.39	130.91	47.01	129.30	2.50	11.00	10609.00	5992.00
ENSMUSG00000038037	Socs1	487.62	538.13	159.49	206.37	60.32	124.91	55.28	208.53	24.88	11.66	1784.75	2112.00	2596.67	3844.00
ENSMUSG00000053113	Socs3	484.52	458.44	417.63	455.86	67.84	87.22	39.03	67.06	534.58	788.26	1327.50	1021.50	3515.00	11969.67
ENSMUSG00000024401	Tnf	3321.70	4880.01	939.77	2661.18	522.03	1837.24	163.15	1196.23	12713.94	16190.19	4658.50	4404.25	41631.00	38744.00

Data are extracted from reported RNA-seq experiments; BMDM, reference (9); BMDC, reference (53); peritoneal neutrophils, (60): reference, T cells; reference (66).

BMDM, bone marrow-derived macrophages.

BMDM, bone marrow-derived dendritic cells.

ND, not determined (too low expression).

In summary, these findings implicate that cell type-specific RBPs act together with TTP to stabilize or destabilize select TTP targets. Comprehensive biochemical studies including reconstitution assays are needed to precisely determine the underlying mechanisms. The results of these studies will be relevant for the entire TTP family, as the functional *versus* silent binding to target mRNA is important also for Zfp36l1 and Zfp36l2 (21, 95).

## Outlook

Despite more than 25 years of research, TTP continues to represent an important and fruitful model for studies on RBPs in general and on the regulation of immune responses by RBPs in particular. Technological progress in recent years and advanced animal models were instrumental for the identification of novel regulatory facets and functional consequences of TTP which fundamentally improved our understating of physiological and pathological inflammation. Most of these new findings remain mechanistically poorly defined and represent challenging topics for future research. This will include analyses of TTP-containing protein complexes and yet uncharacterized phosphorylation sites which will help addressing the mechanism of functional *versus* silent binding of TTP to RNA. An underexplored area are tissue- and cell type-specific functions of TTP *in vivo* to answer the still incompletely understood phenotype of TTP-deficient mice. Finally, an attractive avenue is the exploitation of TTP and mRNA decay in therapy of inflammatory diseases and cancer.

## Author Contributions

PK designed the concept and wrote the manuscript. AB wrote the manuscript and prepared figures. JF prepared figures. All authors contributed to the article and approved the submitted version.

## Funding

This work was supported by the Austrian Science Fund (FWF) grants P33000-B, P31848-B and W1261 to PK.

## Conflict of Interest

The authors declare that the research was conducted in the absence of any commercial or financial relationships that could be construed as a potential conflict of interest.

## Publisher’s Note

All claims expressed in this article are solely those of the authors and do not necessarily represent those of their affiliated organizations, or those of the publisher, the editors and the reviewers. Any product that may be evaluated in this article, or claim that may be made by its manufacturer, is not guaranteed or endorsed by the publisher.
